# Predicting mitophagy-related genes and unveiling liver endothelial cell heterogeneity in hepatic ischemia-reperfusion injury

**DOI:** 10.3389/fimmu.2024.1370647

**Published:** 2024-04-17

**Authors:** Bochen Pan, Xuan Ma, Shihuan Zhou, Xiaoling Cheng, Jianwei Fang, Qiuyun Yi, Yuke Li, Song Li, Jiawei Yang

**Affiliations:** ^1^ Department of Biochemistry, Zunyi Medical University, Zunyi, Guizhou, China; ^2^ Department of Hepatobiliary and Pancreatic Surgery, Affiliated Hospital of Zunyi Medical University, Zunyi, Guizhou, China; ^3^ Department of Cell Biology, Zunyi Medical University, Zunyi, Guizhou, China

**Keywords:** hepatic ischemia reperfusion injury, immune infiltration, endothelial cells, mitophagy, bioinformatic analysis

## Abstract

**Background:**

Hepatic Ischemia-Reperfusion Injury (HIRI) is a major complication in liver transplants and surgeries, significantly affecting postoperative outcomes. The role of mitophagy, essential for removing dysfunctional mitochondria and maintaining cellular balance, remains unclear in HIRI.

**Methods:**

To unravel the role of mitophagy-related genes (MRGs) in HIRI, we assembled a comprehensive dataset comprising 44 HIRI samples alongside 44 normal control samples from the Gene Expression Omnibus (GEO) database for this analysis. Using Random Forests and Support Vector Machines - Recursive Feature Elimination (SVM-RFE), we pinpointed eight pivotal genes and developed a logistic regression model based on these findings. Further, we employed consensus cluster analysis for classifying HIRI patients according to their MRG expression profiles and conducted weighted gene co-expression network analysis (WGCNA) to identify clusters of genes that exhibit high correlation within different modules. Additionally, we conducted single-cell RNA sequencing data analysis to explore insights into the behavior of MRGs within the HIRI.

**Results:**

We identified eight key genes (FUNDC1, VDAC1, MFN2, PINK1, CSNK2A2, ULK1, UBC, MAP1LC3B) with distinct expressions between HIRI and controls, confirmed by PCR validation. Our diagnostic model, based on these genes, accurately predicted HIRI outcomes. Analysis revealed a strong positive correlation of these genes with monocytic lineage and a negative correlation with B and T cells. HIRI patients were divided into three subclusters based on MRG profiles, with WGCNA uncovering highly correlated gene modules. Single-cell analysis identified two types of endothelial cells with different MRG scores, indicating their varied roles in HIRI.

**Conclusions:**

Our study highlights the critical role of MRGs in HIRI and the heterogeneity of endothelial cells. We identified the macrophage migration inhibitory factor (MIF) and cGAS-STING (GAS) pathways as regulators of mitophagy’s impact on HIRI. These findings advance our understanding of mitophagy in HIRI and set the stage for future research and therapeutic developments.

## Introduction

Hepatic ischemia-reperfusion injury (HIRI) is a pathologic scenario characterized by intensified liver damage following the reinstatement of blood flow after a period of ischemia. This phenomenon is a prevalent clinical challenge encountered during significant liver surgeries, liver transplant procedures, and instances of liver trauma (1–4). Commonly, HIRI leads to liver injuries, elevates the risk of early transplantation failure, potentially progresses to liver failure, and in severe cases, may culminate in multi-organ failure (5). Therefore, HIRI remains a significant factor affecting the long-term prognosis of patients undergoing hepatic surgery. Although literature reports various pre- and postoperative treatment methods to alleviate HIRI, these approaches have been effective only in a minority of patients with longer ischemic periods and minimal liver resection (6, 7). Furthermore, as of now, there are no pharmacological treatments officially approved for either preventing or managing HIRI. Researches have suggested that certain interventions, including calcium channel blockers, adenosine receptor agonists, energy metabolism modulators, and antioxidants, could potentially confer protection against HIRI (7, 8). Nonetheless, the considerable adverse effects associated with these treatments have confined their examination largely to animal studies. This absence of robust clinical evidence significantly obstructs their integration into clinical settings (8). Consequently, the quest for developing efficacious drugs or targets to prevent and treat HIRI continues to be a critical area of focus for medical research.

The pathogenesis and development of HIRI are highly complex, resulting from a combination of factors including autophagy, inflammatory cytokines, reactive oxygen species (ROS), calcium overload, energy metabolism disorders, Kupffer cells, complement factors, and non-coding RNAs (9, 10). In recent years, an increasing body of research has confirmed mitochondria as the central link between cellular apoptosis and oxidative stress, highly sensitive to ischemia and hypoxia. When the liver undergoes ischemia-reperfusion injury, the dysregulation of mitochondrial quality control primarily causes cell apoptosis (11–13). Recent studies have highlighted the significance of uncoupling protein 2 (UCP2) as a critical factor in regulating mitochondrial quality control. Overexpression of UCP2 upregulates the deacetylase enzyme 3 (SIRT3) protein, ameliorating mitochondrial damage, suppressing cell apoptosis, and reducing ischemia-reperfusion injury in the heart and brain (14–16). The integrity and equilibrium of mitochondrial function, crucial for sustaining normal energy, oxidant, and metabolic balance, are frequently compromised during HIRI (17–19). Damage to mitochondria leads to the excessive production and build-up of cellular ROS, triggering apoptosis through direct attacks on cellular molecules and fostering the infiltration of inflammatory cells. In HIRI, mitochondrial impairment diminishes ATP production and oxygen consumption during both ischemic and reperfusion phases, adversely affecting cellular energy metabolism and cell survival (20–22). Given the high mitochondrial content, hepatocytes are particularly susceptible to oxidative stress and mitochondrial damage (23).

Autophagy, a universally preserved cellular defense mechanism, plays a pivotal role in maintaining *in vivo* homeostasis. This process entails the breakdown of long-lived proteins and malfunctioning organelles via the formation of autophagosomes (24). As a specialized form of autophagy, mitophagy is crucial for ensuring mitochondrial integrity. This process is regulated by PTEN-induced putative kinase 1 (PINK1) and Parkin, both of which accumulate on the mitochondrial membrane and contribute to the stabilization of mitochondrial depolarization (25, 26). Mitophagy plays a vital role in the progression of liver diseases. In the case of hepatitis B virus (HBV) infection, mitophagy-associated protein upregulation facilitates the degradation of HBx protein aggregates through the ubiquitin-proteasome system (27, 28). On the contrary, the suppression of PINK1/Parkin-mediated mitophagy has been considered an anti-tumor mechanism associated with various Chinese herb extracts (such as matrine and alantolactone). This inhibition promotes apoptosis in HepG2 cells (29, 30). Increasing evidence suggests that reperfusion adversely affects mitophagy, leading to a scenario where the need for mitophagy in removing swollen and damaged mitochondria surpasses the autophagic capabilities of reperfused hepatocytes (31). In the context of HIRI, this deficiency in mitophagy is linked to mitochondrial dysfunction, disruption in oxidation-phosphorylation coupling, and eventual cellular demise (32). Nevertheless, the involvement of mitophagy in HIRI remains less extensively investigated. Recently, next-generation sequencing, utilized at both bulk and single-cell levels, has revolutionized the identification of disease-associated variants, significantly propelling biomedical research forward (33–35). Consequently, we aim to employ this established technology to investigate the involvement of mitophagy in HIRI.

**Figure 1:**
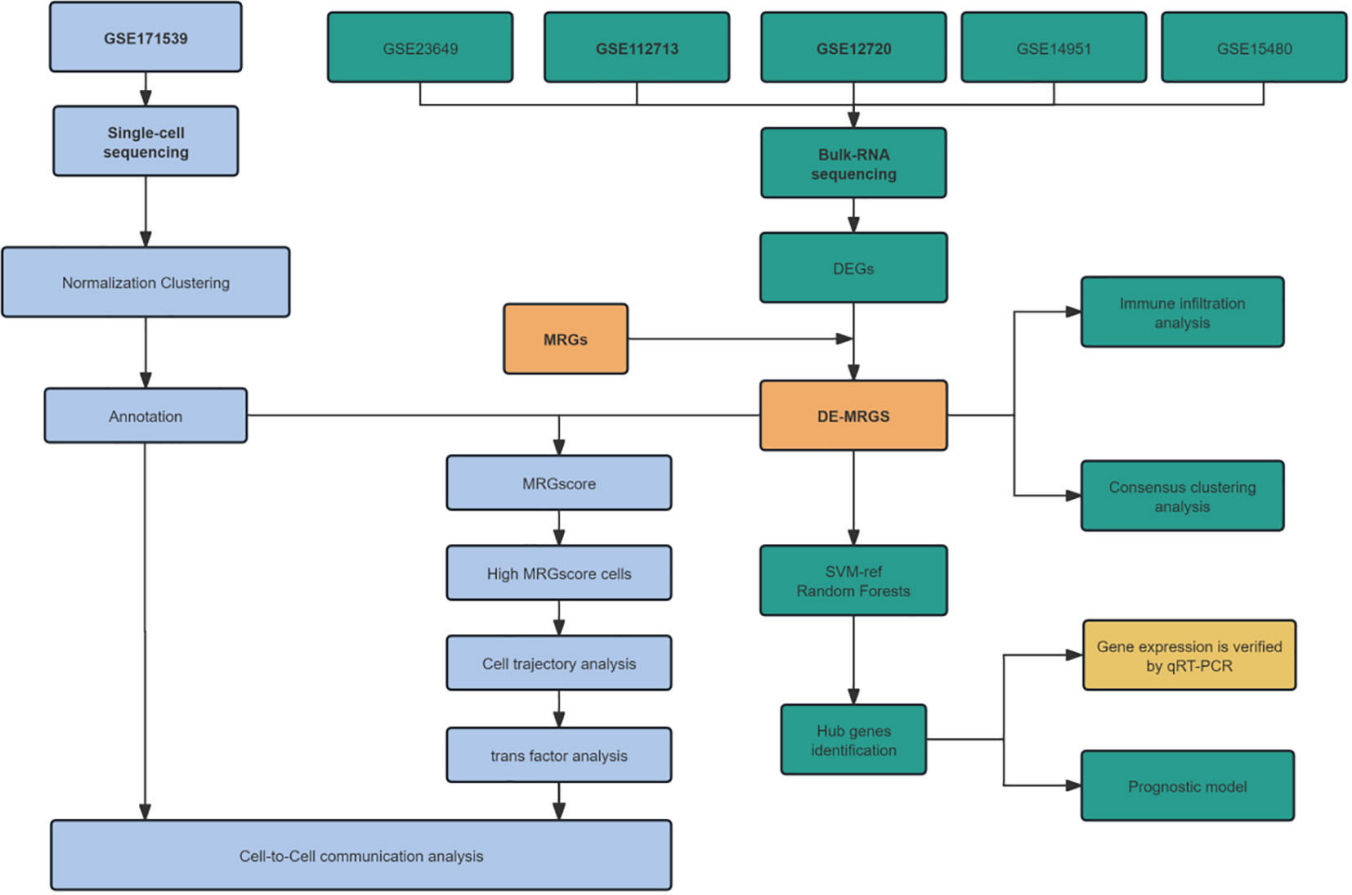
Flowchart for prediction of mitophagy related genes (MRGs) in hepatic ischemia reperfusion injury (HIRI) using integrated bioinformatic analysis and experimental validation.

In this study, we harness the power of bulk and single-cell RNA sequencing data, alongside fundamental molecular biological techniques, to investigate the role of mitophagy-related genes (MRGs) in the pathogenesis of HIRI, as illustrated in **Figure 1**. Specifically, our objective is to decipher the impact of MRGs’ differential expression across various cell types on the severity and variability of the disease, assessing their potential as therapeutic targets for HIRI. This methodology is anticipated to refine diagnostic and treatment strategies, ultimately enhancing the clinical management and prognosis of HIRI patients.

## Materials and methods

### Identification of MRGs expression between HIRI and healthy control group

We selected five gene expression datasets related to HIRI—GSE23649, GSE112713, GSE12720, GSE14951, GSE15480—from the Gene Expression Omnibus (GEO) database (https://www.ncbi.nlm.nih.gov/geo/), using the keyword “Liver ischemia-reperfusion injury” as the basis for our search and selection criteria. The detailed information of datasets is in **Table 1**. A consolidated database comprising 44 HIRI samples and 44 healthy controls was established by performing de-batch operations using the ‘sva’ package in R software. We identified 29 MRGs from Reactome (https://reactome.org/), and their overall expression differences between HIRI and control groups were subsequently analyzed using the ‘limma’ package.

**Table 1:** Liver transplant patient transcriptome data set details.

GEO accession	Platform	Organism	Type	Pre-ischemic sample (n)	Post-reperfusion sample (n)	Reperfusion Time (h)	Age range
GSE23649	GPL6947	*Homo sapiens*	Transplants	9	9	2	NA
GSE112713	GPL14951	*Homo sapiens*	Transplants	11	11	1	NA
GSE12720	GPL570	*Homo sapiens*	Transplants	13	13	1	NA
GSE14951	GPL570	*Homo sapiens*	Transplants	5	5	2-3	40-60
GSE15480	GPL6244	*Homo sapiens*	Transplants	6	6	1.5	30-60
GSE171539	HiSeq X Ten	*Homo sapiens*	Transplants	1	1	2	NA

NA, not available.

### Identification of hub MRGs in HIRI

For pinpointing the pivotal genes in HIRI, we employed random forest classifiers and SVM Recursive Feature Elimination (SVM-RFE) analyses, utilizing the ‘randomForest’ and ‘SVM-RFE’ packages in R (36, 37). Each MRG was assigned a ranking based on its importance as determined by the random forest analysis and the SVM-RFE algorithm, with the top 10 genes being shortlisted. Subsequently, the intersection of these top 10 MRGs, as ranked separately by the random forest and SVM-RFE analyses, was taken to identify the crucial MRGs.

### Construction of receiver operating characteristic curve and nomogram

The identified hub genes were analyzed using multivariate logistic regression, and the ‘Proc’ package was utilized to calculate the area under the receiver operating characteristic (ROC) curve (AUC), assessing their diagnostic efficacy in HIRI. We developed a nomogram to predict HIRI occurrence and plotted calibration and decision curve analyses to demonstrate the model’s robustness. The model underwent internal validation through bootstrap resampling with 1000 iterations. The model’s discriminative ability was gauged using the AUC of the ROC curve, with an AUC exceeding 0.80 being deemed acceptable. We assessed model calibration using the Hosmer–Lemeshow goodness-of-fit test, and the clinical utility of the logistic regression model was evaluated through decision curve analysis (DCA).

### Analysis of relationship between the hub MRGs and immune cells infiltration

To conduct an in-depth immune infiltration analysis, we employed the IOBR package (38). This sophisticated tool facilitated an exploration of the interplay between our selected hub MRGs and a variety of immune cell types, including dendritic cells (DC), mast cells, and CD8^+^ T cells. In addition, we utilized the MCP counter technique for further immune infiltration examination (39). This approach allowed us to quantitatively assess the presence of immune infiltrates, based on gene expression profiles, thus shedding light on the connections between the hub MRGs and specific immune cells such as neutrophils, monocytes, and myeloid cells. Moreover, we performed a correlation analysis to evaluate the relationships between the eight diagnostic MRGs and various inflammatory markers, using the Pearson correlation coefficient to measure the strength and direction of these associations.

### Consensus clustering analysis and co-expression analysis

We executed consensus cluster analysis using the ‘ConsensusClusterPlus ‘package, setting the maximum number of clusterable genes in HIRI samples to 10. For this, the top 5000 most variably expressed genes were determined and used to categorize HIRI samples based on median absolute deviation. Unsupervised consensus clustering was then conducted to assort HIRI samples, identifying the optimal number of clusters (40). Additionally, the Weighted Gene Co-expression Network Analysis (WGCNA) package in R (41) was employed to pinpoint groups of genes that display similar mRNA expression patterns across HIRI samples, utilizing the default settings. The scale-free topology (SFT) criterion guided our selection of the soft threshold parameter for the power adjacency function, with the ideal threshold value being determined according to the SFT criterion recommendation, ensuring a model-fit saturation greater than 0.85.

### GO, KEGG and GSVA function analysis

We employed the Molecular Signatures Database (MSigDB) to obtain gene sets pertinent to Homo sapiens within the ‘hallmark’ category, utilizing the ‘msigdbr’ package in R for this purpose. Additionally, GO (Gene Ontology) and KEGG (Kyoto Encyclopedia of Genes and Genomes) pathway analyses were performed using the ‘clusterProfiler’ package (42) in R, GO, and Kyoto Encyclopedia of Genes and Genomes (KEGG) pathway analyses were also conducted. Metascape (43) was also used for further enrichment analysis. For gene set variation analysis (GSVA), we opted for the ‘GSVA’ package in R, selecting the single-sample gene set enrichment analysis (ssGSEA) approach with a Gaussian kernel cumulative distribution function for our computations (44).

### Basic analysis workflow of scRNA-seq data

In our single-cell RNA sequencing (scRNA-seq) data quality control process, we retained cells that expressed at least 300 genes and exhibited mitochondrial gene counts below 20% of total gene counts. Using the CellCycleScoring function in Seurat package (version 4.3.0) (45), each cell was categorized into a specific cell cycle stage. For data normalization, we applied the scTransform function, incorporating both S. Score and G2M.Score. Dimension reduction and clustering analyses were carried out using the Seurat package. To address the batch effect, which might compromise the precision of single-cell analyses, we employed the harmony package (version 0.1.1) for batch effect correction, focusing on the top 3000 variable genes with the default harmony parameters. The selection of principal components (PCs) was guided by elbow and Jackstraw plots. Clustering was performed using FindNeighbors and FindClusters functions in Seurat, which utilize a shared nearest neighbor (SNN) modularity optimization-based clustering algorithm on the selected PCs with a resolution setting of 0.5. UMAP visualizations were generated using Seurat’s RunUMAP function, with clusters being identified based on unique gene expression profiles. The MRGs score was calculated using UCell V.2.2.0 (46). For differential gene expression analysis, we used the FindMarkers function with default settings. A similar approach was followed for the secondary clustering of endothelial cells, maintaining a resolution of 0.5.

### Construction and analysis of the transcription factor-gene network

We employed pySCENIC (47, 48) to identify transcription factor regulons. A count matrix was generated, focusing on 10,000 variable genes. Genes expressed in less than 1% of cells were excluded in accordance with the pySCENIC protocol recommendations. The gene co-expression network was established using the gradient boosting machine within Arboreto. Predicted enriched motifs for each gene co-expression module were obtained through precomputed databases from cisTargetDB and pySCENIC’s ctx function. Finally, AUCell was utilized to quantify the activity scores of inferred regulons at the single-cell level.

### Cell-cell communication analysis

For the analysis of cell-to-cell communication, we utilized the R CellChat package (v 1.6.1) (49). Initially, the normalized expression matrix was imported into the CellChat object using the ‘createCellChat’ function. Subsequently, default parameter preprocessing steps were executed, including functions like ‘identifyOverExpressedGenes,’ ‘identifyOverExpressedInteractions,’ and ‘projectData’ Potential ligand-receptor interactions were calculated using functions such as ‘computeCommunProb,’ ‘filterCommunication’ (min.cells = 10), and ‘computeCommunProbPathway.’ Finally, the cell communication network was consolidated using the ‘aggregateNet’ function.

### Cell culture and group processing

Normal human hepatocytes (HL7702, L02) were procured from the Cell Bank of the Chinese Academy of Sciences (Shanghai, China) and subjected to rapid thawing for revival. Subsequently, the cells were cultured in RPMI-1640 medium supplemented with 100 U/mL penicillin, 100 μg/mL streptomycin, and 10% fetal bovine serum. This culture was maintained at 37°C in a 5% CO_2_ incubator. Following three stable passages, the cells were divided into two groups: the normal L02 cell group (NC) and the hypoxia/reoxygenation (HIRI) group. When cell confluence reached 70%-80%, they were subjected to a 6-hour hypoxic pre-acclimatization period in a three-gas hypoxic incubator (Thermo, MA, USA). During this period, cells were cultured in sugar-free Dulbecco’s modified Eagle medium with a gas mixture consisting of N_2_ (94%), O_2_ (1%), and CO_2_ (5%). The durations of hypoxia (6 hours) and reoxygenation (12 hours) treatments were determined based on the study by Huang et al. (50).

### Real-time quantitative PCR (RT-qPCR) assay

Total RNA was extracted from the cells using RNAiso Plus (Takara, Tokyo, Japan), quantified, and subjected to reverse transcription to obtain cDNA. The target mRNA was subsequently amplified through a standard two-step PCR procedure, utilizing a reaction volume of 25 µL, which included 12.5 µL of TB Green Premix Ex Taq II, 1 µL of specific forward primer, 1 µL of specific reverse primer, 2 µL of cDNA, and 8.5 µL of sterile water. Following amplification, the relative expression levels of the genes were determined using the 2^-ΔΔCq^ method and normalized against β-actin. The primer sequences for the target genes are provided in **Table 2**.

**Table 2:** Experimentally relevant primers.

Gene name	Forward Primer	Reverse Primer
h-MFN2	CTCTCGATGCAACTCTATCGTC	TCCTGTACGTGTCTTCAAGGAA
h-CSNK2A2	TCCCGAGCTGGGGTAATCAA	GTTCCACCACGAAGGTTCTCC
h-MAP1LC3B	AAGGCGCTTACAGCTCAATG	CTGGGAGGCATAGACCATGT
h-PINK1	GCCTCATCGAGGAAAAACAGG	GTCTCGTGTCCAACGGGTC
h-ULK1	GGCAAGTTCGAGTTCTCCCG	CGACCTCCAAATCGTGCTTCT
h-UBC	CCTGGTGCTCCGTCTTAGAG	TTTCCCAGCAAAGATCAACC
h-VDAC1	ACGTATGCCGATCTTGGCAAA	TCAGGCCGTACTCAGTCCATC
h-FUNDC1	CCTCCCCAAGACTATGAAAGTGA	AAACACTCGATTCCACCACTG

### Statistical analysis

All raw data processing was conducted using R software (version 4.2.1). To identify significant differences between two independent groups, either Student’s t-test or Wilcoxon’s rank sum test was applied, depending on the data distribution. For comparisons involving more than two independent groups, the Kruskal-Wallis test was employed. All p-values were calculated as two-sided, and statistical significance was established at *p* < 0.05.

## Results

### Identification of mitophagy-related differentially expressed genes in HIRI

**Figure 2:**
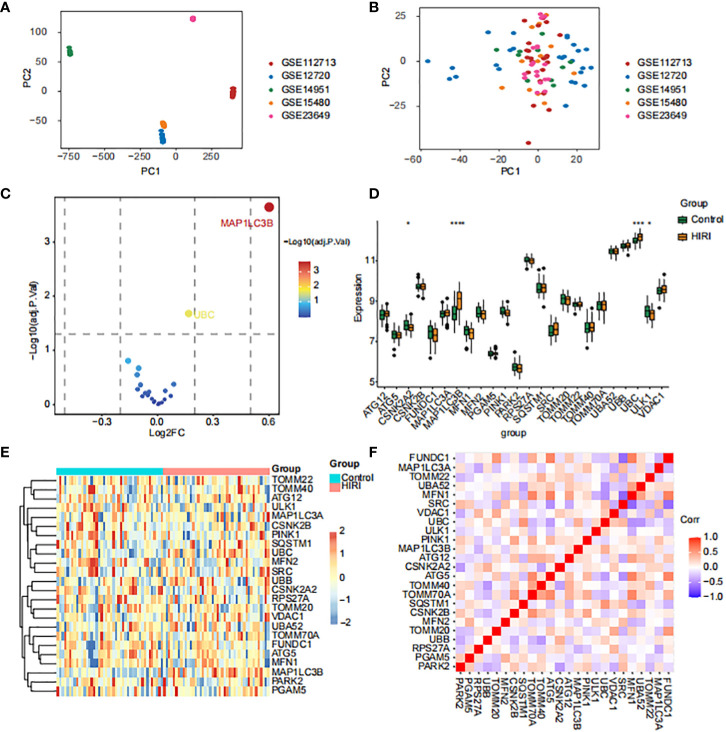
Identification of mitophagy-related differentially expressed genes (MRDEGs) in HIRI. **(A, B)** The principal component analysis (PCA) graph of five obtained datasets before **(A)** and after **(B)** batch removing. **(C–F)** The volcano plot **(C)**, box plot **(D)**, heat map **(E)** and correlation heat map **(F)** analysis of MRDEGs expression. Statistical significance was assessed using the Wilcoxon rank-sum test to compare scores. *p < 0.05, ***p < 0.001, ****p < 0.0001 vs. normal control.

The datasets GSE23649, GSE112713, GSE12720, GSE14951, and GSE15480 underwent processing using the “sva” R package to address batch effects and facilitate comprehensive data integration. This process resulted in the selection of 44 control subjects and 44 cases with HIRI. The principal component analysis (PCA, **Figures 2A, B**) illustrates the successful elimination of batch effects. To explore inter-group mitophagy related differentially expressed genes (MRDEGs), we effectively utilized the linear models for microarray and RNA-seq data (limma) approach. We identified 24 genes that exhibited differential expression; the depiction of MRDEGs’ expression patterns across diverse groups (**Figures 2C–E**) revealed notable findings. Genes such as MAP1LC3B and UBC showed a significant increase in HIRI expression, while ULK1 and CSNK2A2 exhibited notably reduced expression. Further examination of the correlation among MRDEGs revealed a strong positive correlation between UBC and SQSTM1, contrasting sharply with a significant inverse correlation between UBC and MFN1 (**Figure 2F**).

### Establishment of HIRI diagnostic model based on hub MRGs.

**Figure 3:**
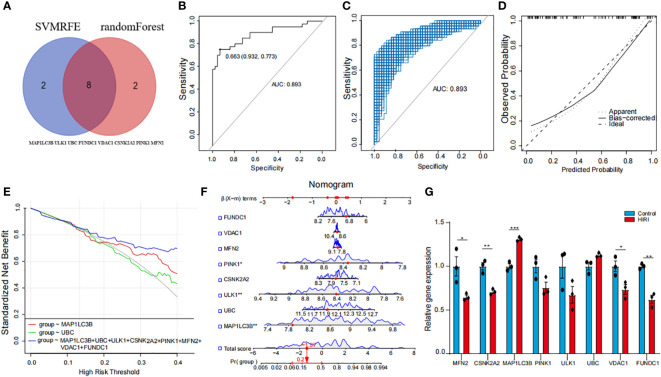
Development and validation of a HIRI diagnostic model using hub MRGs **(A)** Eight hub genes identified by random forest and Support Vector Machines—Recursive Feature Elimination (SVM-RFE) analysis. **(B)** Receiver Operating Characteristic (ROC) curve of predicted risk scores in HIRI diagnosis. **(C)** AUC score calculation and confidence interval estimation using 1000 bootstrap samples **(D)** Calibration curve to assess the predictive power of the logistic model. **(E)** Comparison of decision curve analysis (DCA) of different genes. **(F)** Nomogram of eight hub MRGs in the diagnosis of a HIRI sample. **(G)** qPCR validation of hub genes expression in hepatocyte L02 cells. The methods for statistical analysis were the same as in **Figure 2**. *p < 0.05, **p < 0.01,***p < 0.001, ****p < 0.0001 vs. normal control.

Harnessing the combined strengths of Random Forests and Support Vector Machines—Recursive Feature Elimination (SVM-RFE), we crafted a refined selection strategy for MRGs to delve into their roles in HIRI. This meticulous process led to the discovery of eight key genes: FUNDC1, VDAC1, MFN2, PINK1, CSNK2A2, ULK1, UBC, and MAP1LC3B (**Figure 3A**). Utilizing these genes, we developed a logistic regression model, creating a sophisticated predictive framework for HIRI (**Supplementary Figure 1A**). This model showcased impressive predictive performance (AUC = 0.893), as illustrated in **Figure 3B**. To ensure the Receiver Operating Characteristic (ROC) curve’s accuracy, we employed a bootstrap method, generating random samples (n = 1000 bootstraps) with replacement (**Figure 3C**), and demonstrated the model’s stability with a calibration curve (**Figure 3D**). The comprehensive analysis of AUC, specificity, and sensitivity, presented in **Supplementary Figures 1B–D**, confirms the model’s diagnostic prowess. Through a nomogram, the model offered diagnostic precision exceeding that achievable with individual genes alone (**Figures 3E, F**), thereby improving the accuracy of patient diagnosis. This innovative diagnostic model, grounded in MRGs, highlights their significant impact on HIRI’s progression. Subsequent qPCR validation experiments using human hepatocyte L02 cells to reinforce the predictive accuracy of our model concerning the identified hub genes, as illustrated in **Figure 3G**. The results demonstrated that the majority of genes exhibited the correct trends and were significant in the cell model, with the exception of ULK1, UBC, and PINK1. This variance between the qPCR outcomes and our initial expectations could be due to the distinct gene expression profiles in human liver tissues compared to our *in vitro* cell model. This validation not only confirms the model’s reliability but also highlights the significant potential of these genes as indicators for HIRI progression. Specifically, the L02 cell experiments demonstrated notable changes in gene expression patterns almost consistent with our predictions, providing concrete evidence that supports the model’s effectiveness in identifying critical molecular markers of HIRI.

### Distinguishing mitophagy regulators and immune infiltration patterns among mitophagy clusters

**Figure 4:**
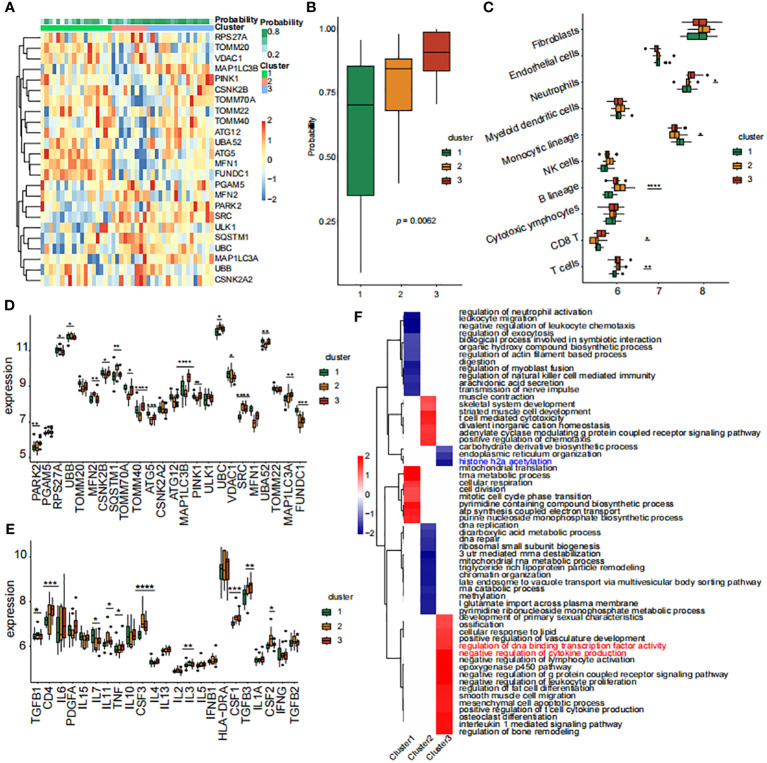
Analysis of mitophagy regulators and immune infiltration across mitophagy clusters. **(A)** The Heat map of the analysis of differences among the three subtypes and correlation analysis between subtypes and clinicopathological features. **(B)** The proportion of tissue grade in the three subtypes. **(C)** The box line plots described the expression levels of immune cells between clusters. **(D)** The box line plots described the mRNA expression levels of mitophagy-related DEGs between clusters. **(E)** The box line plots described the expression levels of inflammatory factors between clusters. **(F)** GSEA pathway differential analysis showed activated and inhibited pathways in the three clusters. The methods for statistical analysis were the same as in **Figure 2**. *p < 0.05, **p < 0.01,***p < 0.001, ****p < 0.0001 vs. normal control.

Building upon the identification of the 8 diagnostic genes, our analysis further explored the complex interplay between mitophagy regulator expression patterns and the immune landscape within HIRI patients. We grouped the patients into three distinct clusters using consensus cluster analysis based on these patterns (**Figure 4A**). Notably, patients in cluster 3 showed a higher likelihood of HIRI diagnosis (**Figure 4B**). Both B lineage and T cell activations were distinctly enriched in cluster 3 (**Figure 4C**), underscoring the role of adaptive immunity in modulating HIRI severity. This suggests an enhanced immune response may serve as a double-edged sword, offering defense but also potentially exacerbating tissue damage. Cluster 3 displayed a significant upregulation of MAP1LC3B, PINK1, MAP1LC3A, SRC, TOMM40, alongside a notable downregulation of FUNDC1 and TOMM20 (**Figure 4D**). Additionally, Differences of inflammatory factor expression revealed elevated levels of CSF1, CSF3, CD4, and TGFB3 in cluster 3 (**Figure 4E**). These all reflected a biological mechanism where disrupted mitophagy and heightened inflammatory responses converge, leading to a more pronounced susceptibility to HIRI. Furthermore, employing GSEA for enrichment analysis, we conducted pathway differential analysis across the three clusters. The outcomes highlighted the activation of pathways associated with “negative regulation of cytokine production” and “negative regulation of lymphocyte activation” within the clusters, indicating a potential compensatory mechanism to mitigate excessive immune activation. Conversely, the inhibition of the histone H2A acetylation pathway could signal alterations in gene expression regulation, affecting immune response and cellular stress mechanisms (**Figure 4F**).

### Gene modules screening and co-expression network construction

**Figure 5:**
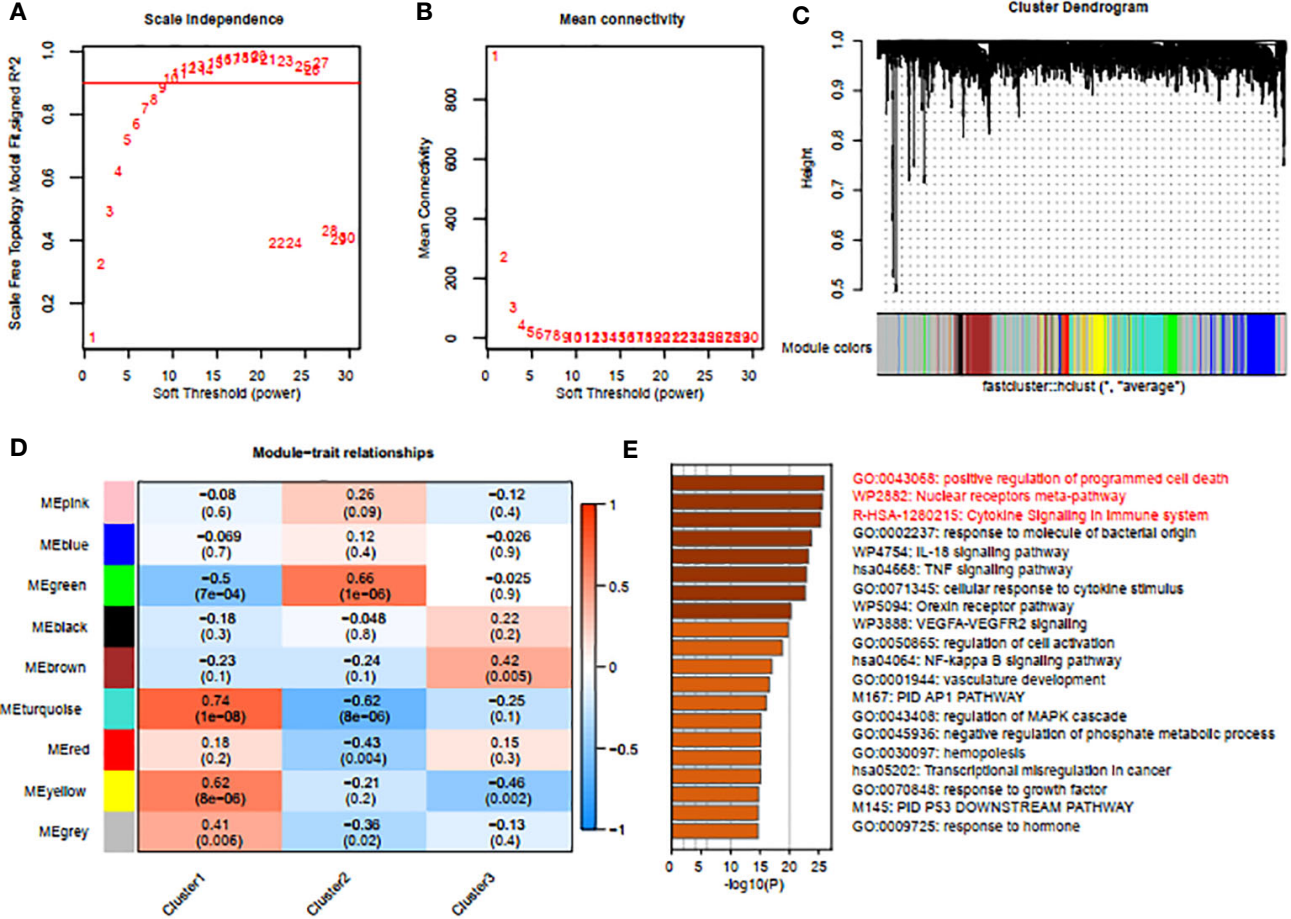
Screening of gene modules and construction of co-expression networks. **(A, B)** Analysis of network topologies for various soft-thresholding powers through the scale-free fit index **(A)** and mean connectivity **(B)**. **(C)** Cluster tree dendrogram of co-expression modules. **(D)** Correlation analysis between module eigengenes and clinical status. Each row represents a module; each column represents a clinical status. **(E)** Enrichment analysis using Metascape for genes in the blue module.

To identify gene modules closely associated with mitophagy clusters, we used WGCNA to examine the correlated genes within the three clusters of interest. Quality control was conducted on all 88 HIRI samples without exclusion. Co-expression modules were established using dynamic tree-cut analysis, setting the soft threshold at 9 to achieve optimal scale-free topology in network construction (**Figures 5A, B**). Significant co-expression modules were identified through optimal dynamic tree-cut and hierarchical clustering methods (**Figure 5C**). Significantly, the brown module displayed a strong positive correlation with cluster 3 (*p* < 0.05, **Figure 5D**). Using Metascape, we conducted enrichment analysis on genes within the brown module, revealing pathways such as “positive regulation of programmed cell death”, “nuclear receptors meta-pathway” and “cytokine signaling in immune system” (**Figure 5E**).

### Hub gene expression linked to immune cells and inflammatory markers

**Figure 6:**
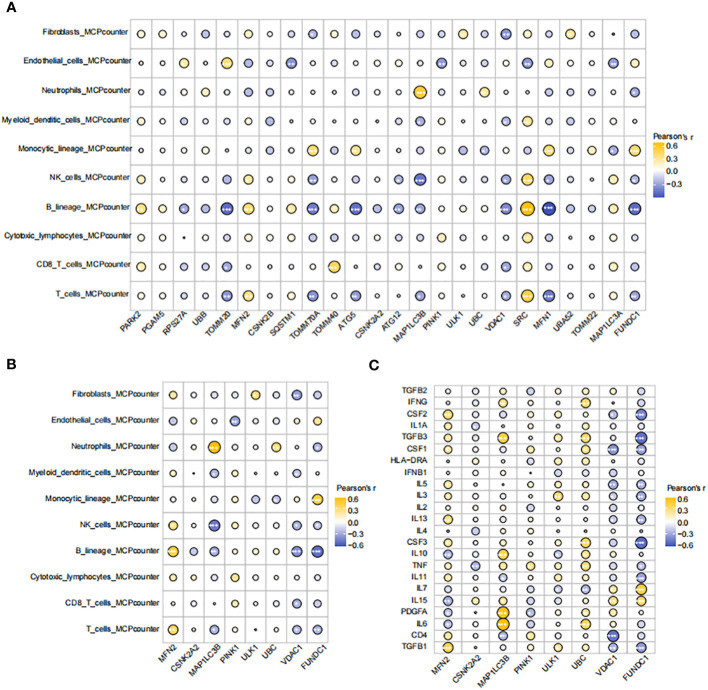
Correlations between hub genes, immune cells, and inflammatory factors **(A)** Correlation map of genes and immune cells based on CIBERSORT algorithm. **(B)** Correlation map of eight diagnostic genes and immune cells based on MCPcounter algorithm. **(C)** Correlation diagram of eight diagnostic genes and inflammatory factors. The size of the circle represents the strength of the pearson correlation coefficient between the different variables. *p < 0.05, **p < 0.01,***p < 0.001, ****p < 0.0001 vs. normal control.

We conducted an immune-cell infiltration analysis using the IOBR package. Among the 24 genes analyzed, MAP1LC3B exhibited a positive correlation with Neutrophils, Mast cells activated, and Monocytes (**Figure 6A**). In contrast, MAP1LC3B showed negative correlations with Mast cells resting and Macrophages M2. Similarly, MFN2 displayed positive associations with T cell CD8 and Macrophages M2 but exhibited a negative association with Monocytes (**Figure 6A**). Furthermore, utilizing the MCP-counter method, the diagnostic genes demonstrated robust correlations with B lineage, Neutrophils, NK cells, T cells, and Monocytic lineage (**Figure 6B**). An assessment of the connections among the eight hub MRGs and inflammatory factors indicated strong correlations between the eight hub genes and inflammatory factors such as TGFB1, TGFB3, IL7, TNF, IL3, IL6, PDGFA, IL10, and CSF2 (**Figure 6C**).

### Single-cell analysis of the Expression of MRGs in HIRI.

**Figure 7:**
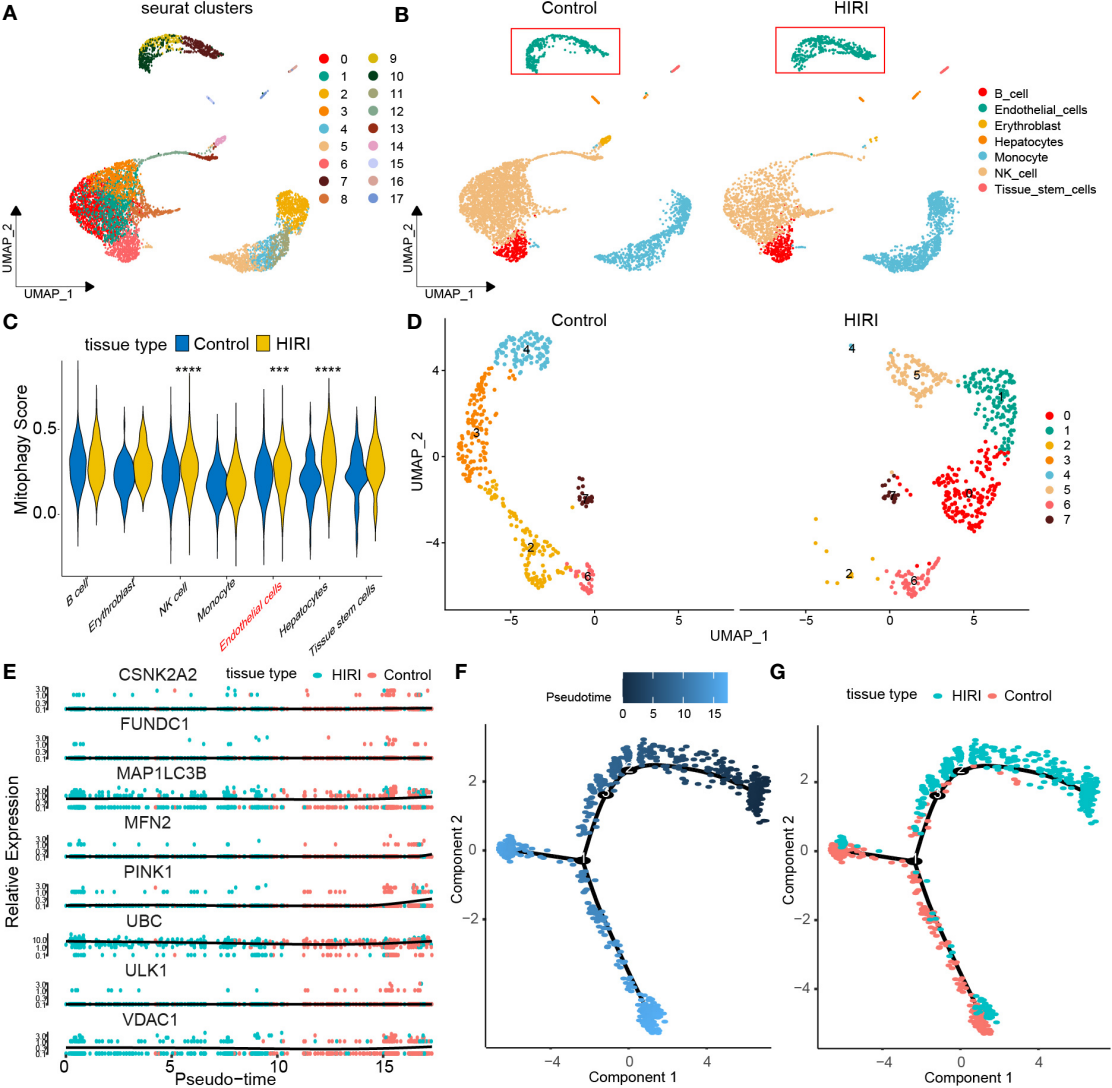
Dissecting endothelial MRG expression in HIRI through single-cell analysis. **(A)** Cell clusters for GSE171539 of two HIRI samples. **(B)** Cell markers for clusters’ annotation. **(C)** the mitophagy score between HIRI and control. **(D)** UMAP plot depicting eight endothelial cells types. **(E)** Pseudotime trajectory inferred by monocle and colored according to tissue type. **(F)** Monocle-based pseudotime trajectory colored by pseudotime. **(G)** Monocle-based pseudotime trajectory colored by tissue type. The methods for statistical analysis were the same as in **Figure 2**. ***p < 0.001, ****p < 0.0001 vs. normal control.

To enhance our understanding of how mitophagy-related genes impact HIRI, we explored the GSE171539 dataset, which comprises single-cell sequencing data from liver transplants, allowing us to assess mitophagy activities at the single-cell resolution. Initially, cell populations were classified into 14 clusters (**Figure 7A**). Subsequently, these clusters were categorized into seven distinct cell populations: B cells, endothelial cells, erythroblasts, hepatocytes, monocytes, NK cells, and tissue stem cells, based on marker gene expression. We observed a novel population of endothelial cells in the HIRI group, which was distinct from those present in the control group (**Figure 7B**). Extensive analysis also unveiled noteworthy variations in mitophagy scores between the HIRI and control groups among endothelial cells (**Figure 7C**). Pursuing this, we performed further dimensionality reduction and clustering on endothelial cells, which highlighted the conspicuous changes and marked heterogeneity when comparing HIRI with the control group (**Figure 7D**). Building on this, our subsequent pseudo-temporal analysis mapped out a developmental trajectory resembling a tree-like structure, showcasing different tissue states. During the initial and middle phases of this trajectory, there was a dominant presence of HIRI-related cells and gene expressions within endothelial cells. Yet, as the trajectory advanced toward later stages, these expressions gradually shifted to resemble those of normal tissue more closely. This progression led us to validate the developmental pathway of key genes within endothelial cells, further corroborating our findings regarding the tissue developmental trajectory (**Figures 7E–G**).

### Cell communication analysis between endothelial cells with varying degrees of mitophagy and other cells

**Figure 8:**
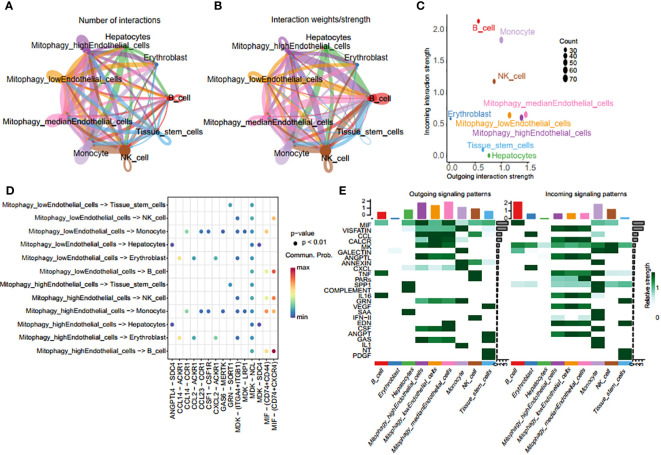
Crosstalk analysis of endothelial cells with diverse MRG scores and monocytes. **(A, B)** Graphic representation of intercellular communication dynamics across various cell types. **(C)** Comparison of signal transduction and reception capabilities between mitophagy high endothelial cells and mitophagy low endothelial cells. **(D)** Heatmap of ligand-receptor pairs of different mitophagy score endothelial cells with other cell types. **(E)** Heatmap of all signaling pathways between all cell types.

Endothelial cells were classified into three categories based on their MRG scores: mitophagy high endothelial cells (scoring above the 75^th^ percentile), mitophagy low endothelial cells (scoring below the 25^th^ percentile), and mitophagy median endothelial cells (scoring within the 25^th^ to 75^th^ percentile). Using CellChat, we examined intercellular communication variations among endothelial cells with different MRG scores and other cell types. Particularly, mitophagy high endothelial cells demonstrated heightened communication with monocytes (**Figures 8A, B**), depicted graphically to illustrate intercellular communication dynamics among diverse cell types. Mitophagy high endothelial cells showed significant potential for both signal transduction and reception, whereas mitophagy low endothelial cells primarily exhibited high signal reception ability (**Figures 8C, D**). The results revealed a preferential utilization of the macrophage migration inhibitory factor (MIF) and cGAS-STING (GAS) signaling in communication between mitophagy high and low endothelial cells (**Figure 8E**).

### Scenic analysis of transcriptional factor profiles in high mitophagy endothelial cells and low mitophagy endothelial cells

**Figure 9:**
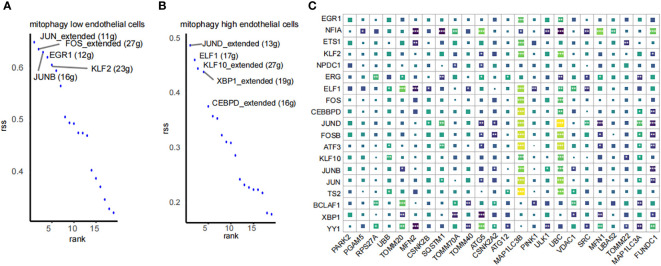
SCENIC analysis of transcription factor profiles in endothelial cells by mitophagy level. **(A, B)** Transcription factor profiles in mitophagy high endothelial cells and mitophagy low endothelial cells. **(C)** Correlation analysis between the top 10 transcription factors unique to high and low MRG score endothelial cells and MRGs. *p < 0.05, **p < 0.01,***p < 0.001, ****p < 0.0001 vs. normal control.

Using pySCENIC, we investigated the differences in transcription factors between two types of endothelial cells displaying varying MRG scores. Transcription factors significantly associated with low mitophagy scores included FOS (+), GR1 (+), JUNB (+), KLF2 (+), while those closely linked with high mitophagy scores comprised ELF1 (+), KLF10 (+), XBP1 (+), and CEBPD (+) (**Figures 9A, B**). Additionally, we performed a correlation analysis at the bulk transcriptome level between the top 10 unique transcription factors from each group and MRGs. The results indicated that the top 10 transcription factors associated with high mitophagy scores are mostly positively correlated with MRGs (**Figure 9C**). These findings emphasize the pivotal role of these transcription factors in regulating mitophagy in HIRI.

## Discussion

HIRI represents a complex pathophysiological phenomenon in which various factors collaborate to exacerbate liver damage, dysfunction, and structural injury upon the restoration of blood flow following periods of inadequate or interrupted circulation to the liver (51). This condition not only exerts a detrimental impact on liver function, potentially resulting in dysfunction or failure in affected individuals, but also triggers a robust stress and inflammatory response. This response can, in turn, induce acute dysfunction in other vital organs such as the heart, kidneys, and lungs, occasionally culminating in acute multi-organ failure (52). HIRI is not a minor complication; rather, it is a severe condition with profound systemic repercussions that significantly influence the prognosis and quality of life of patients (53). Consequently, the matter of mitigating or preventing the adverse effects of HIRI remains a prominent topic in clinical practice, yet effective solutions have not been fully realized.

Mitophagy, involving the selective degradation of mitochondria through autophagy, is integral to cellular homeostasis. It upholds the quality of the mitochondrial network by eliminating damaged or surplus mitochondria (54). Given the unclear definition of mitophagy’s role in HIRI, we conducted comprehensive bioinformatics analysis and experiments to explore their relationship theoretically. In the current era, the rapid advancements in scRNA-seq technology provide an innovative approach for investigating HIRI (55). In this current study, we have undertaken a thorough analysis of MRGs in HIRI. This analysis encompasses both bulk and single-cell RNA sequencing data analysis, allowing for a comprehensive exploration of the role of mitophagy in the HIRI.

By analyzing bulk RNA sequencing data, we discovered a potential significant role of MRGs in HIRI. Initially, we identified eight pivotal genes—FUNDC1, VDAC1, MFN2, PINK1, CSNK2A2, ULK1, UBC and MAP1LC3B—with distinct expression patterns in HIRI patients compared to controls. Leveraging these key genes, we established an effective diagnostic model for HIRI. PINK1, recognized as a multifunctional protein among the hub MRGs, holds a crucial role in cellular processes including autophagy and mitophagy, which involve the selective degradation of mitochondria (56). MFN2, located in the outer mitochondrial membrane, assumes a critical role in facilitating mitochondrial fusion and actively participates in essential physiological processes. These processes encompass mitochondrial autophagy, the formation of structural connections between mitochondria and the endoplasmic reticulum, as well as apoptosis. Its involvement significantly influences the onset and progression of HIRI (57). Furthermore, we observed that the expression of hub genes aligned consistently with our analytical outcomes in qPCR validation. This concordance in qPCR validation further bolsters the reliability of our analysis.

Moreover, our results provide substantial evidence for the link between the expression of key MRGs and the immune microenvironment, as well as inflammatory factors. Specifically, we observed a strong positive correlation between the eight central MRGs and monocytic lineage, in contrast to a noteworthy negative correlation with B lineage and T cells. This underscores the intricate interplay between mitophagy and the immune response in the HIRI (58). Additionally, we uncovered a robust correlation between colony stimulating factors and MRGs, highlighting the significant influence that colony stimulating factors may exert on mitophagy in HIRI. Lastly, our analysis suggests that HIRI can be categorized into three subtypes based on MRGs expression patterns, revealing substantial heterogeneity in MRGs expression across these three clusters. These findings could have significant implications for patient stratification and the development of personalized treatment strategies in the HIRI.

Our extensive examination of MRGs and the heterogeneity of endothelial cells in HIRI at the single-cell level enriches our comprehension of this intricate inflammatory condition. Notably, we identified a significant disparity in mitochondrial autophagy scores within endothelial cells between HIRI and normal conditions. Studies have investigated mitophagy’s role in endothelial cells, especially under conditions of oxidative stress and limited energy supply (59–61). endothelial cells with damaged mitochondria are removed by mitophagy, which lessens cellular damage (62, 63). Moreover, exposure of endothelial cells to hemin initiates a cascade of lipid peroxidation, leading to mitochondrial depolarization and subsequent activation of mitophagy (64).

Our cell trajectory analysis indicated that genes associated with MRGs, expressed initially in damaged tissue during HIRI, contribute to a protective role. The analysis of intercellular communication revealed notable differences between endothelial cells with high and low MRGs scores. Endothelial cells with high mitophagy activity primarily engaged in communication via the MIF signaling pathway, whereas endothelial cells with low mitophagy activity displayed reduced communication through the GAS signaling pathway. The MIF pathway exerts a significant influence on HIRI by modulating liver inflammation, apoptosis, and oxidative stress. Elevated levels of MIF in HIRI patients suggest its involvement in liver injury (65). The GAS pathway assumes a multifaceted role in the pathogenesis of HIRI, impacting tissue remodeling, inflammation, immune regulation, and mucociliary clearance. Disruption of this pathway may exacerbate HIRI symptoms, highlighting its potential as a therapeutic target (66). These findings indicate the involvement of both the MIF and GAS pathways in mediating the effects of mitophagy in HIRI. Additionally, distinct transcription factor profiles were identified between endothelial cells with high and low mitophagy activity. A significant negative correlation was observed between the prominent transcription factor, ELF1(+), and MRGs in endothelial cells with high mitophagy activity, suggesting its significant role in regulating mitophagy.

This research represents a pioneering effort to dissect the complex interplay between endothelial dysfunction and mitophagy in the HIRI, employing an innovative combination of bulk and single-cell RNA sequencing alongside experimental validation. This multifaceted approach has provided us with detailed insights into the molecular underpinnings of HIRI, uncovering previously unrecognized dimensions of the condition and offering a comprehensive view of the endothelial alterations associated with it. Such insights have the potential to reshape our understanding of HIRI’s pathophysiology, paving the way for novel diagnostic and therapeutic strategies that target these newly identified pathways. We acknowledge certain limitations within our study, such as the representativeness of our sampled data and the reliance on RNA sequencing for drawing our conclusions. We propose that future research should incorporate proteomic analyses and functional assays to validate and extend our findings, thereby offering a more robust foundation for the development of targeted interventions. Our investigation into the significant role of MRGs and the heterogeneity of endothelial cells in HIRI marks a significant advancement in the field. By identifying and analyzing key MRGs, we have illuminated their crucial roles in modulating immune responses and inflammation within the HIRI context. Such findings not only enhance our understanding of HIRI’s molecular landscape but also offer new avenues for patient stratification and the development of tailored treatment strategies. The consistent expression patterns of hub genes observed in qPCR validation further validate our analytical approach, while the diverse MRGs scores noted at the single-cell level suggest a nuanced involvement of endothelial cells in HIRI’s pathogenesis. Importantly, our study underscores the regulatory importance of the MIF and GAS pathways in the context of mitophagy’s impact on HIRI. This highlights potential therapeutic targets that could be exploited to mitigate the effects of HIRI, suggesting a promising direction for future research and clinical applications. In conclusion, our comprehensive exploration of HIRI not only contributes to a deeper understanding of the condition’s molecular basis but also sets the stage for the development of innovative therapeutic approaches. By bringing to light the critical roles of MRGs and uncovering the complex interactions within HIRI’s pathophysiological framework, our study offers a valuable resource for advancing both scientific research and clinical practice in the management of HIRI.

## Data availability statement

The original contributions presented in the study are included in the article/**Supplementary Material**. Further inquiries can be directed to the corresponding author.

## Ethics statement

Ethical approval was not required for the studies on humans in accordance with the local legislation and institutional requirements because only commercially available established cell lines were used.

## Author contributions

BP: Formal analysis, Investigation, Methodology, Writing – original draft, Resources. XM: Data curation, Investigation, Writing – original draft, Validation. SZ: Formal analysis, Investigation, Software, Writing – original draft. XC: Investigation, Funding acquisition, Writing – review & editing. JF: Software, Writing – original draft, Investigation. QY: Software, Writing – original draft, Formal analysis. YL: Investigation, Writing – original draft, Software. SL: Investigation, Writing – original draft, Software. YS: Formal analysis, Writing – original draft. JY: Conceptualization, Funding acquisition, Supervision, Writing – review & editing, Validation.

## Supplementary material

Supplementary Table 2:1 List of DE-MRGs in HIRI.
