# Supplementary material for: Predicting mitophagy-related genes and unveiling liver endothelial cell heterogeneity in hepatic ischemia-reperfusion injury
Source: Front Immunol. 2024 Apr 17;15:1370647. doi: 10.3389/fimmu.2024.1370647 (PMC11061384; doi:10.3389/fimmu.2024.1370647)
Supplement: Supplementary Table 2:1 — List of DE-MRGs in HIRI. [file DataSheet_1.docx]

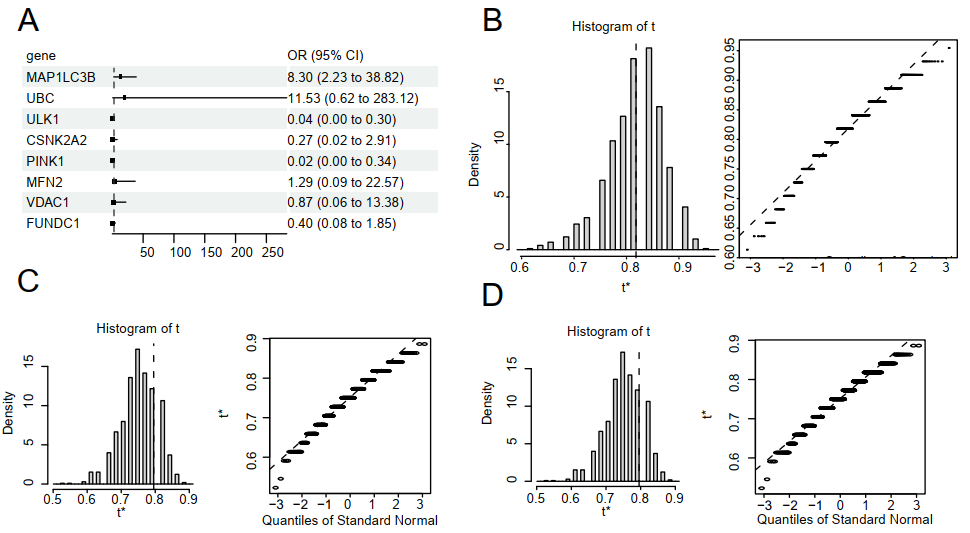


**Fig. S1** Indicators for successful diagnostic model establishment. (A) OR value and confidence interval of key genes. (B) Distribution range of under area of AUC. (C) Distribution range of sensitivity. (D) Distribution range of specificity.
